# Gynecology

**Published:** 1876-02

**Authors:** 


### VI. Gynecology.

1. *Paralysis from a Retained Pessary.* Dr. Gervis, London Obstetrical Society. (*Med. & Surg. Reporter*, January, 1876.)

At a late meeting of the Obstetric Society of London, Dr. Gervis exhibited a pessary which had been removed from a patient fifty-six years of age. Fifteen years previously, it had been introduced in the vagina to relieve prolapsus of the womb. Four years ago she began to feel a weakness in the lower back. Two years ago her legs became weak, and she was unable to walk without assistance. For six months she has been bedridden. For the last six months she has had discharge from the vagina, occasionally tinged with blood, and highly offensive. She can neither stand nor walk, and is able to move her legs in bed with difficulty, being attacked two or three times during the night with cramp in them. On examination, a large round metallic pessary was found imbedded in the vaginal walls, and was removed by the aid of bone forceps, a considerable quantity of brownish purulent discharge, horribly offensive, escaping. All the paralytic symptoms were relieved after its removal. Dr. Cleveland remarked that, apart from the interest attaching to the long impaction of a foreign body in the vagina, there was the notable fact that a quantity of highly offensive putrid matter must have been locked up for a considerable time, without the supervention of septicæmia. The case tended to show, in connection with the recent debate before the Society, the necessity for the co-existence of some peculiar condition of the general system before absorption of morbid matter, followed by blood-poisoning, could be effected.

2. *Cases Showing the Importance of Making a Careful Physical Examination.* By Clement Godson, M.D., Assistant Obstetric Physician to St. Bartholomew's Hospital. (*The Obstet. Jour.*, Dec., 1875.)

Case I.—On August 14th, 1875, C. N., single, aged seventeen, of excessively anæmic appearance, applied at the out-patient room, stating that she had been losing large quantities of blood from the vagina since Christmas; had been regular from fourteen years of age up to that time; had never missed a period. Cannot say that she has been free a day since Christmas; is sometimes deluged with blood, and passes large clots. Previously never suffered from menorrhagia or leucorrhœa.

The under-garments, even the petticoats, were found saturated with blood. The abdomen showed no signs of enlarged uterus. The cervix uteri was ascertained by vaginal examination to be short, the os patulous the uterus retroverted, but freely movable.

The patient was admitted into the hospital.

For two or three weeks, complete rest, with the cold douche, and the administration, first of ergot and subsequently of cannabis indica in large doses, also the replacement of the uterus by means of a pessary, were tried, but with little or no improvement. On September 6th, some cotton wool saturated with tr. ferri. perchlor. was passed into the uterus. It seemed to grate over a roughened surface towards the fundus. This only served to increase the hæmorrhage.

On September 14th, the os uteri having previously been well dilated by tents, I passed my finger into the uterus, and detached from the fundus a quantity of soft pulpy tissue, having very much the appearance of decidua. From this day the hæmorrhage ceased entirely, and the patient left the hospital on October 12th, perfectly recovered.

CASE II.—On October 7th, 1875, S. E., aged forty-seven, was led into the out-patient room, supported by two friends, walking in a doubled-up position, the face expressive of great suffering. She stated that she had been married twenty-one years, and had had eight children and five miscarriages, the last, a child, seven years ago. Catamenia commenced at thirteen years of age, and continued with regularity up to three years ago, excessive in quantity during the last ten years. No appearance for the last three years. Has been subject to leucorrhœa ever since marriage. Three weeks ago was seized with violent pain in lower back and abdomen, which has never abated, and prevents her from sleeping. This is accompanied by a discharge from the vagina, of a brownish color, sometimes sanious, and very fetid.

No abdominal swelling was detectable. A vaginal examination showed the cervix to be very short, smooth and indurated, the uterine walls to be felt bulging from it on all sides, the os patent, and a quantity of sloughing tissue protruding from it. Uterus quite movable.

The patient was admitted into the hospital, and in the evening a sponge tent was inserted.

On the following day I introduced my finger into the uterus, the os being well dilated, and with the aid of the ovum forceps I succeeded in removing all the sloughing mass which I could feel attached to the posterior wall. The odor from this was very fetid, and I washed out the uterus with a weak solution of iodine. A few days subsequently a slight attack of pelvic cellulitis occurred, and some deposit was detected posterior to the cervix. This, however, soon cleared up. The uterus is now quite movable, the patient is free from discharge and pain, and her general health is rapidly being restored.

CASE III.—On October 6th, 1875, E. B., aged thirty-two, applied at the out-patient room, her face very sallow and expressive of pain.

Has been twice married, thirteen years ago to first husband, by whom she had two children; eight months since to present husband. Catamenia regular till five months ago, up to which time was quite well. Since then there has been a continual discharge from the vagina, which is either like pure blood, or is of a brownish color, and is very fetid; sometimes passes large clots.

*Physical Examination.*—Roof of vagina transformed into an irregular mass; the os uteri very patulous; in the centre of it the tissue in some parts very dense, in others friable, breaking down and bleeding when touched. The hand on abdomen comes upon a well-defined swelling, reaching almost to the umbilicus, pressure upon which is directly conveyed to finger in vagina. On abdominal auscultation the fœtal heart can be plainly detected. *Mammæ* very attenuated; turbid serum can readily be squeezed from nipples.

The patient was admitted into the hospital.

On visiting the ward on October 11th, I was informed that during the night a large quantity of watery fluid had suddenly passed from the vagina, and the patient had been in intense agony ever since. She was rolling in the bed with pain; her pulse almost imperceptible, and so rapid it could hardly be counted. I found the os uteri dilated as widely as seemed possible without the tissues being rent, and the fœtal head was presenting. The patient was at once placed under the influence of ether, and I perforated the head, and subsequently the chest, and succeeded in extracting a fœtus (as far as I could judge, of nearly six months' development) through quite a small aperture, without having exerted any undue force in traction upon it. The placenta soon followed, and there was hardly any loss of blood.

She gradually sank, and died on the 13th from exhaustion. The autopsy showed no signs of a rent in the uterine structure.

*Remarks.*—These cases show the importance of a careful physical examination. In the first there was no history of pregnancy, or even coitus, ever having taken place; a vaginal examination was insufficient to discover the cause of the hæmorrhage, and it became necessary to explore the cavity of the uterus before the origin was ascertained. In the second case the symptoms were rather those of carcinoma, and the vaginal examination alone revealed the real nature of the disease, a submucous fibroid sloughing. In the third case carcinoma of the cervix was believed to exist, and the patient had been treated for such by her medical attendant, but no idea of pregnancy existed. There was no history to lead to such supposition. It was the examination of the abdomen alone which led to the discovery. It shows, therefore, the importance of extending the investigation further than a mere vaginal examination.

3. *Note on the Discharge of Ova, and its Relation in Point of Time to Menstruation.* Dr. Jno. Williams, M.D. (*The Obstet. Jour. of Great Britain and Ireland*, Dec., 1875.)

The exceeding great value of all observations made on this subject by physicians, capable of properly investigating it, is the only reason for giving so much space to it. The reporter, Dr. Williams, assistant obstetric physician to University College Hospital, London, read this paper before the Royal Society of London, last May.

It is a recognized fact in physiology that ova are discharged in connection with the menstrual function, but it is uncertain at what time in the course of the month the separation takes place. It is generally understood to occur towards the end of the discharge, or immediately after its cessation. I have, however, reason to believe, from observations made in several subjects, that such is not the case, but that it takes place before the appearance of the monthly flow with which it is connected. The cases which have come under my observation fall into four series, as follows:—

*A. Cases, six in number, in which a Graafian follicle had been matured and actually ruptured.*

(1). The first of these was a young girl who died through the effects of a fall, three or four days before the expected return of the catamenia. In the left ovary was a recently ruptured Graafian follicle. The cavity of the follicle was about $\frac{3}{4}$ inch in diameter, and contained a recent clot, which projected slightly through the rupture ; the clot was of a fresh red color, nowhere adherent to the parts around, for on making a section through the follicle it fell out. The wall of the vesicle was of a pale yellowish color, and slightly wrinkled. The rupture had evidently taken place a short time only before death.

(2). The second subject was a woman who died suddenly through a fall, probably a fortnight after the cessation of the last menstrual flow. On examination a considerable quantity of blood was found in the cavity of the peritoneum, and the liver was torn. In the left ovary was a ruptured follicle, with corrugated and collapsed walls; its cavity contained no blood, but there was a slight effusion between its lining membrane and its outer coat. The depth of the follicle from the rupture to the furthest point of the opposite wall measured nearly $\frac{3}{4}$ inch. It is not impossible that this follicle was ruptured somewhat prematurely by the shock of the fall.

(3). The next example was observed during life. Mr. Christopher Heath performed ovariotomy on a patient on the fourteenth day after the cessation of the last catamenial discharge. Menstruation lasted usually three days, and the patient had always been regular every four weeks. In the ordinary course of things the next flow would have appeared in eleven days. When the diseased ovary had been removed, the remaining one was raised up, with a view to see if it were healthy, and it was observed that it contained an enlarged Graafian follicle, which became ruptured when being held in the hand. I ought to add that the flow returned three days after the operation, and eight days before it was due.

(4). The next case was a young woman who died of pleurisy on the fifth day of menstruation. On the surface of the left ovary was a rough, brownish-colored, star-like cicatrix. On section, there was seen under the cicatrix, a corpus luteum, dilated in the middle and narrow at both ends, nearly $\frac{3}{4}$ inch in length and $\frac{1}{2}$ inch in width; its walls were in some parts of a pinkish and in others of a yellowish color, slightly, if at all, thicker than those previously mentioned, and had small prominences on its inner surface. In the centre was a partially decolorized clot, which was but slightly adherent to the surrounding walls. From these characters it is evident that the rupture of the follicle had taken place several days before.

(5). The fifth member of this series was a patient who died on the fourth day of menstruation, and about the ninth of typhoid fever. One ovary contained a corpus luteum similar to the one just described.

(6). The last example occurred in a young girl, who died of pneumonia six days after the cessation of the catamenia. On the surface of the right ovary was a small cicatrix, beneath which was a corpus luteum with the following characters :—It was of an irregular, elongated shape, nearly $\frac{1}{2}$ inch in length and $\frac{1}{4}$ in width; had thick, yellow, convoluted walls, and enclosed a small whitish mass, in which were two dark-colored spots, which were evidently the remains of a clot. This ovary contained also a Graafian follicle of the size of a small pea. The determination of the age of effused blood is always difficult. In the Graafian follicle which becomes ruptured without impregnation taking place, it is known that certain definite changes occur ; the wall of the vesicle becomes thick, yellow, and convoluted ; the blood which flowed into it and filled it becomes decolorized and absorbed. The exact length of time in which these changes in the follicle are brought about, is not accurately determined ; but it is known that the corpus luteum of one menstruation has become considerably atrophied by the return of the next.

It appears to me that the yellow body in the last example of this group was considerably older than the two preceding ones, and that it was more than a fortnight old, and that the two preceding ones were from eight to ten days.

B. *Cases, four in number, in which a Graafian follicle had been matured and hæmorrhage had taken place into its cavity, but no actual rupture had occurred.*

(1). The first case was a patient who died of pyæmia in the third week after the cessation of the last catamenial flow. The left ovary contained a follicle $\frac{3}{4}$ inch in diameter, distended by a recent non-adherent, softish coagulum, uniform in consistence and color. This follicle was prominent above the adjacent surface of the ovary; and its superficial wall was thick, and presented no tendency to point or rupture. There was no recent rupture to be seen on the surface of either ovary.

(2). The second example was a woman who had undergone an operation for fistula in ano. The monthly flow made its appearance a week before the expected time for its return, and she died five days after. One ovary contained a follicle measuring $\frac{5}{8}$ inch by $\frac{1}{8}$ inch; this follicle contained a bright red, fresh, loose clot, and its walls were thin and not corrugated. From these characters it appears that the hæmorrhage into the follicle had taken place but a short time before death.

(3). The next was a patient who had undergone an operation for the removal of an ovarian tumor. She died a fortnight after the operation, when she had menstruated for one day. At the inner extremity of the left ovary was a large, dark-colored, softish mass, which, on section, proved to be a Graafian follicle containing a brick-red-colored clot, which appeared to be of a spongy texture. It could with difficulty be turned out of the sac. After its removal it was seen that the wall of the sac was formed by a thin yellowish substance.

(4). The last example in this group was a person who suffered with fibroid tumor of the uterus. She died on the third or fourth day of the menstrual flow. Both ovaries were bound to the surrounding structures by tough and firm false membranes. The left contained a follicle nearly an inch in length, in which was found a softish, dark-colored clot, having a spongy texture, which appeared to be several days old.

In the first and third members of this group hæmorrhage had taken place into the follicle unquestionably before the appearance of the catamenial discharge.

In the second, hæmorrhage had occurred before the flow had become due; but the latter, owing to surgical interference, having returned a week before its time, the hæmorrhage took place while the discharge was in progress.

In the fourth, the condition of the clot makes it almost certain that the hæmorrhage had taken place before the appearance of the catamenia.

C. *One case, in which a Graafian follicle had matured, but where neither rupture nor hæmorrhage had actually occurred.*

This was a patient who died of typhoid fever just before the appearance of the catamenia. In one ovary there was an enlarged Graafian follicle, which was highly vascular, and projected like a nipple beyond the surrounding surface. It was evidently on the point of bursting, and it is doubtful whether rupture of the follicle or the appearance of the discharge would have first taken place.

D. *Cases, three in number, in which no Graafian follicle had become enlarged to the size exhibited by it at maturity.*

The first was a patient in whom the menstrual flow had almost ceased. There was no rupture in either ovary, but the right contained a Graafian follicle about the size of a small pea.

The next was a young suicide, who died three days after the cessation of the catamenial discharge. There was no recent rupture in either ovary, but the left contained a follicle similar to the one seen in the preceding case.

The last member of the series was a girl who died of peritonitis, caused by the rupture of an abscess on the right ovary. In the left was a Graafian follicle about the size of a small pea, but no recent rupture. The state of the lining membrane of the uterus showed that in this case menstruation was imminent.

Besides the appearances described, there were in all the preceding cases numerous Graafian follicles, varying in size from a millet-seed downwards, together with some superficial pits and atrophied corpora lutea.

These cases appear to me to bear out the opinion stated at the beginning of this paper, that, in the great majority of subjects, the discharge of ova takes place before the appearance of the menstrual flow with which it is connected; for in ten out of the fourteen, rupture of a follicle, or hæmorrhage into its cavity, had occurred before the return of the catamenia; in one it was doubtful whether rupture of a follicle, or the appearance of the discharge would have taken place first; in two a menstrual period had passed without maturation of a follicle; and in one a periodical discharge was imminent, though the ovaries contained no matured Graafian follicle. It is not improbable that the follicles which were found in the last three cases, and which were enlarged to the size of a small pea, would have become mature by the next return of the flow.

I have carefully considered the cases recorded by Cruikshank, Jones, Paterson, Lee, Girdwood, Negrier, Coste, and others, and find that, though they do not contribute materially to the solution of the question discussed in this paper, yet, in so far as they go, they favor the view put forward here—a view which derives support from the custom imposed by the Levitical law, and observed to this day by the stricter sect of the Hebrew community.

POSTSCRIPT.

Since writing the above, I have had opportunities to examine two subjects in whom the date of the last menstruation was known.

The first was a girl, aged seventeen years, who died on the fifth day after admission to the Middlesex Hospital, of traumatic tetanus. She was said to have ceased to menstruate just before admission; and the condition of the inner surface of the uterus confirmed that statement. The uterus and ovaries were small and imperfectly developed. On the surface of the right ovary was found a patch ½ inch in diameter, slightly injected, and presenting a punctated appearance. In its centre was a cicatrix, appearing as a white spot, beneath which was situated a yellow body, elongated and irregularly flattened in shape. This appeared to be due to pressure from several Graafian follicles growing in close proximity to it, the largest of which was as large as a small pea. The yellow body measured nearly ½ inch in length; it had folded walls, and in its centre was a thin elongated clot, the middle of which was of a dark color.

The second subject was aged twenty-six years; she died of Bright's disease. The last menstruation began May 13th, ceased May 19th, and death occurred May 28th, fifteen days after the appearance of the flow. Hæmorrhage had taken place into the superficial tissue of the ovaries, probably by reason of the condition of the blood.

In the right was a small superficial prominence formed by a yellow body, which measured about ⅜ inch in diameter; it was throughout of a yellowish color, and contained no trace of the coloring matter of blood. On comparing these organs with one another and with those previously described, I am led to infer that in the first, 12 to 14 days, and in the second about 20 days, had elapsed since rupture of the follicle occurred.

Reichert has examined twenty-three organs in which signs of menstruation were recognizable. In four cases a Graafian follicle had matured but not ruptured, nor had hæmorrhage taken place, though the decidua menstrualis was in a state of greater or less development; in eighteen cases a Graafian follicle had ruptured, and hæmorrhage had taken place into the decidua; in one case only, in which bleeding had not begun, had a Graafian follicle been ruptured. The latter statement appears opposed to the conclusions at which I have arrived; but this is only apparent; for in one case a follicle had ruptured, in four a Graafian follicle had matured before hæmorrhage began, and in one of these, rupture was on the eve of taking place; in eighteen, a follicle had ruptured, and hæmorrhage had taken place into the decidua menstrualis. Put in this form, Reichert's cases are not opposed to the conclusions arrived at in the preceding note; and as his cases have not been described, it is not possible to say what their actual bearing may be. The conclusion arrived at by Reichert, after examination of the twenty-three specimens, however, is, that rupture of the Graafian follicle takes place at an early stage of the menstrual flow.

4. *Early Menstruation.* ANON. (*Brit. Med. Jour.*)

"M.D." writes: I have now under my observation, a girl aged two years, who has menstruated regularly for some time. She is in perfect health, and her courses, which are in every respect natural in color, occur every month, causing her much discomfort and pain.

5. *Menstruation and the Law of Monthly Periodicity.* Prof. J. GOODMAN, M.D. (*Richmond and Louisville Med. Jour.*, Dec., 1875.)

The writer so admirably concludes his long article, that we cannot refrain from placing before our readers, in his own words, at length, the pretty definitely settled conviction of the profession at the present day on this vexed question:

The law of monthly periodicity is that which governs and determines the periodic return of those phenomena which collectively constitute menstruation, and must necessarily be the result and exponent of certain changes or series of changes that transpire within the organism in recurring cycles; these cycles being uniform in their duration, definite in their manifestations, and common to woman-kind. The essence or factor of the law, then, is a dynamic cycle of physiological acts. But in what part of the system does this cycle revolve? Where is the seat of these changes?

Not in the ovaries, as Tilt supposed, for their annihilation does not always arrest the action of the law.

Nor in the uterus, for women in whom this organ has been congenitally absent, have experienced unmistakable signs of monthly periodicity.

Paracelsus, DeGraff, and others located the process in the blood. Nature has negatived this class of theories by presenting us with the Hungarian Sisters, in whom the bloodvessels were united at the loins, thus giving them a common circulation, but their menstrual periods were different.

Tyler Smith has advanced an ingenious hypothesis, according to which the monthly cycle is attributed to reciprocal action between the uterus, ovaries, and mammæ. But, as we have seen, two of these supposed agents may be withdrawn with impunity.

An affirmative answer to this question is just as easy and may be just as absolute as those in the negative. There is not the slightest doubt that the functional process by which the monthly cycle is accomplished is seated in the ganglionic nervous system. The expansion of the pelvis, the growth of the breasts, and the increase of adipose tissue which take place when the young girl arrives at the age of puberty, depends upon a modification in the nutrition of the parts, and that nutrition throughout the body is presided over by the ganglionic nerves, no one at the present day will deny. In order to achieve such important variations from the previous course of nutrition, the nervous tissue itself must undergo structural development, which, as we have already said, like all other structural alterations, has a tendency to be permanent. In the course of this development, the elements of the law of periodicity are elaborated, and the series of changes constituting the monthly cycle are instituted, the operation of the law being usually declared through the circulation (by means of hæmorrhages), which, like nutrition, is controlled by the ganglionic system.

This is the limit to which inductive reasoning will carry us at present, and it is better to await the results of observations than indulge in vague conjectures. What the nature of the changes is, and in what part of the sympathetic system these occur, I leave to anatomists and experimental physiologists to discover.

The only practical bearing of these views to which I will allude at present, is with reference to the operation of 'Normal Ovariotomy.' The originator of this operation, Dr. Battey, anticipated that it would bring about an artificial menopause. In this he will certainly be disappointed in at least half the cases, but it does not seem to me absolutely essential to the success of the operation that it should arrest the progress of the law of monthly periodicity and put a stop to menstruation. That the ovaries continue, after puberty, to affect the general system, and that this influence is even necessary in many individuals to maintain the sexual development in a perfect degree, is undoubtedly a fact, as is shown by an appreciable loss, in some instances, of sexual traits, including the cessation of menstruation upon the removal of these organs. In diseased conditions of the ovaries, this reaction is perverted, and very distressing pathological states, especially of the nervous system, arise in consequence. Such examples are frequently encountered in practice, and a conclusive, though an anomalous, one has been referred to in a former part of this article. It is in severe and intractable cases of this kind that good results are to be hoped for from the operation alluded to; for it is reasonable to believe that when disordered action arises from an unnatural irritant, a withdrawal of the irritant will permit the functions to return to their normal channels.

